# Fermentation characteristics and microbial community composition of wet brewer’s grains and corn stover mixed silage prepared with cellulase and lactic acid bacteria supplementation

**DOI:** 10.5713/ab.23.0177

**Published:** 2023-08-16

**Authors:** Guoqiang Zhao, Hao Wu, Yangyuan Li, Li Li, Jiajun He, Xinjian Yang, Xiangxue Xie

**Affiliations:** 1 Guangdong VTR Bio-Tech Co., Ltd., Zhuhai, 519060, China; 2 State Key Laboratory of Animal Nutrition, College of Animal Science and Technology, China Agricultural University, Beijing, 100193, China

**Keywords:** Cellulase, Corn Stover, Fermentation Quality, Lactic Acid Bacteria, Microbial Community, Wet Brewer’s Grains

## Abstract

**Objective:**

The objective of this study was to investigate how cellulase or/and lactic acid bacteria (LAB) affected the fermentation characteristic and microbial community in wet brewer’s grains (WBG) and corn stover (CS) mixed silage.

**Methods:**

The WBG was mixed thoroughly with the CS at 7:3 (w/w). Four treatment groups were studied: i) CON, no additives; ii) CEL, added cellulase (120 U/g fresh matter [FM]), iii) LAB, added LAB (2×10^6^ cfu/g FM), and iv) CLA, added cellulase (120 U/g FM) and LAB (2×10^6^ cfu/g FM).

**Results:**

All additive-treated groups showed higher fermentation quality over the 30 d ensiling period. As these groups exhibited higher (p<0.05) LAB counts and lactic acid (LA) content, along with lower pH value and ammonia-nitrogen (NH_3_-N) content than the control. Specifically, cellulase-treated groups (CEL and CLA) showed lower (p<0.05) neutral detergent fiber and acid detergent fiber contents than other groups. All additives increased the abundance of beneficial bacteria (Firmicutes, *Lactiplantibacillus*, and *Limosilactobacillus*) while they decreased abundance of Proteobacteria and microbial diversity as well.

**Conclusion:**

The combined application of cellulase and LAB could effectively improve the fermentation quality and microbial community of the WBG and CS mixed silage.

## INTRODUCTION

With the current livestock industry development, the shortage of high quality feed is well recognized. Thus, the efficient utilization of unconventional agricultural byproducts to achieve sustainable and economically viable livestock systems has attracted increasing interest. Wet brewer’s grains (WBG), which can be widely obtained in China, is the most important and abundant brewing process byproduct generated by the brewery industry. WBG have high moisture content, as its 93% water and only 7% solids. It contains abundant digestible fibrous materials and has a high nitrogen-containing composition, normally 23% to 27% crude protein (CP) of the dry matter (DM) basis [1]. Therefore, it may be a potential feed resource for ruminants. However, due to its high moisture content, it is difficult for transportation and long-term preservation. The high transportation cost and quick deterioration thereby potentially limits its utilization as a common feed resource. Multiple studies have shown that mixing high moisture byproducts with dry crop byproducts is a solution for achieving favorable fermentation quality and reducing problems related to high moisture materials [2,3]. Hence, it is necessary to find a proper byproduct which is cost-effective and has a high biomass yield. Corn (*Zea mays* L.) stover, as one of the most abundant agricultural byproducts with high lignocellulosic biomass, is widely used around the world. China reportedly produces approximately 220 million tons of dry corn stover annually, with the majority being directly incinerated as waste, thereby wasting feed resources, and causing serious environmental concerns [4]. Therefore, the demand for efficiently utilizing corn stover is increasing, not only as an eco-friendly approach for corn residue disposal, but also as a suitable ruminant feed resource, especially in places where corn is cultivated as the main crop. However, corn stover generally is moisture-deficient and which is unfavorable to fermentation, with its fibers not easily being degraded by ruminating. Moreover, lackingimportant nutrients like protein makes it difficult to meet the feed requirements of ruminants. Considering all these factors, ensiling the high-moisture WBG with the dry corn stover might be a proper way to enhance the fermentation quality and palatability of both materials nutritionally, eco-friendly, and economically. Using additives is necessary because converting rough forage materials to high quality silage is difficult due to their hard and stemmy structures with high fiber and low carbohydrate content. Lactic acid bacteria (LAB) and cellulase are the two most widely used additives during fermentation. The LAB anaerobically converts water-soluble carbohydrate (WSC) into organic acids, mainly lactic acid, thereby decreasing the silage pH and inhibiting the unfavorable microbes [5]. Similar reports have also shown that LAB addition maintains the dominance of acid producing microbes during the ensiling process, thereby creating a suitable pH environment, inhibiting undesirable microbial activities, and reducing proteolysis [6]. Moreover, LAB accelerates the time required for pH to stabilize during the initial stage of fermentation. Cellulase application increases the substrates available for LAB growth during fermentation by hydrolyzing structural carbohydrates, like the cell wall, into glucose [7]. To date, information about ensiling WBG and corn stover, and their detailed characteristics during fermentation have rarely been reported. Therefore, the current study investigated how cellulase and LAB application affected the fermentation quality and microbial community of the WBG and corn stover (CS) mixed silage.

## MATERIALS AND METHODS

### Silage preparation

The experiment was conducted in the experimental field of Guangdong VTR Bio-Tech Co., Ltd., in the Doumen District, Zhuhai, China (22°8′19″ N, 113°14′6″ E, altitude −7 m). The experimental area was within the subtropical marine climate zone, where the annual average temperature, precipitation, and humidity were 22.3°C, 2,061.9 mm, and 78.7%, respectively. The WBG was purchased from a private beer company (Zhujiang Beer Co., Ltd., Guangzhou, China). The CS byproduct was collected from the whole crop corn after removing the grain, cob, and husk, followed by cutting it into 2 to 3 cm pieces using a hand cutter. Then the WBG was mixed thoroughly with the dry corn stover at 7:3 (w/w). The chemical composition of WBG and corn stover prior to ensiling are shown in Table 1. The mixed materials were treated as follows: i) no additive (CON); ii) added cellulase (CEL; 120 U/g fresh matter [FM], Guangdong VTR Bio-Tech Co., Ltd., Zhuhai, China); iii) added LAB (LAB; 2×10^6^ cfu/g FM, purchased commercial LAB product); and iv) added cellulase and LAB (CLA). The cellulase additive and LAB inoculant were dissolved in distilled water and sprayed on the WBG-CS mixture and then evenly mixed. For the control group, an equivalent volume of distilled water was added.

Approximately 150 g of treated materials were then ensiled into vinyl plastic bags (28×35 cm; Huaguan Printing Co., Ltd., Wenzhou, China) and sealed with a vacuum sealer (Dafeng Machinery Co., Ltd., Wenzhou, China). All the 36 bags (3 ensiling periods×4 treatments×3 replicates) were then preserved at ambient room temperature (around 25°C) for d 7, 14, and 30. The silage samples of 30 d were subjected to chemical composition analysis, and microbial community. Furthermore, samples of 7, 14, and 30 d were taken to analyze the dynamic changes of fermentation characteristics. Three replicates were performed for each treatment.

### Chemical composition analysis

The plastic bags were opened after 30 d of storage and the fermented materials were thoroughly mixed. Three sub-samples were taken for each replicate. Approximately 60 g samples were dried in an air-forced drying oven at 65°C for 48 h to determine the DM content. Subsequently, the dried samples were ground and passed through a 1-mm sieve for chemical analysis. The CP content was determined using the K9860 Kjeldahl Nitrogen Analyzer (Hanon Advanced Technology Group, Jinan, China) according to the guidelines of the Association of Official Analytical Chemists [8]. Neutral detergent fiber (NDF) and acid detergent fiber (ADF) were analyzed via the Van Soest method [9] using an ANKOM 2000 Automated Fiber Analyzer (Ankom Technologies, Fairport, NY, USA). The WSC was analyzed by the modified anthrone-sulphuric acid method [10]. Ether extracts (EE) and crude ash content of raw materials were determined by the AOAC method [8].

### Fermentation characteristics and microbial analysis

Samples of d 7, 14, and 30 were taken and stored at −80°C prior to analysis. Samples (20 g) were homogenized in distilled water (180 mL) at 4°C for 24 h. Thereafter, the pH, NH_3_-N, and organic acids analysis of the silage extract was made by filtering the mixture through four layers of cheesecloth and qualitative filter paper. The pH was measured using a PHS-3C pH meter (Inesa Instrument, Shanghai, China). The NH_3_-N content in the extract was analyzed according to the phenol-sodium hypochlorite colorimetric method [11]. The concentration of organic acids, including lactic acid (LA), acetic acid (AA), propionic acid (PA), and butyric acid (BA) were determined using the Agilent HPLC 1260 (Agilent Technologies, Santa Clara, CA, USA), which was equipped with a 210 nm UV detector (Sciex API 5000; McKinley Scientific, Sparta Township, NJ, USA) and Agilent Hi-Plex H column (Agilent Technologies, USA). The eluent was 5 mM H_2_SO_4_ with a running rate of 0.7 mL/min at a 55°C column oven temperature. Microbial analysis was made using the plate counting method [12]. Samples (10 g) were stirred well in a blender with 90 mL sterilized saline water and were then serially diluted (10^−1^ to 10^−8^). LAB, *Escherichia coli*, viable molds, and yeast were incubated and quantified on de Man, Rogosa and Sharpe (MRS) agar at 30°C for 48 h anaerobically, violet red bile agar at 37°C for 24 h, and potato dextrose agar at 28°C for 48 to 72 h, respectively. Microbiological data were collected as colony-forming units (cfu) and were logarithmically transformed on an FM basis.

### Microbial community

Silage samples of d 30 were used for microbial community analysis using the high-throughput sequencing platform of Novogene Co., Ltd. (Tianjin, China). DNA was extracted from silage samples through the cetyltrimethylammonium bromide method [13]. The concentration and purity of the extracted DNA was evaluated by 1% agarose gel electrophoresis and spectrophotometrically at 260/280 nm ratio. Subsequently, the DNA was diluted by sterile water to 1 ng/μL. Using gene-specific primers, the V3–V4 regions of the *16S rRNA* gene were amplified by polymerase chain reaction (PCR), and then these amplicons were paired-end sequenced on the NovaSeq 6000 platform (Novogene, Beijing, China). Thereafter, effective tags were obtained by assembling raw reads using FLASH (version 1.2.7) and discarding low-quality reads (quality scores <20) according to the QIIME (version 1.9.1) quality control process. The final effective tags were subsequently clustered into operational taxonomic units (OTUs) based on a 97% sequence similarity level. Based on the OTU results, the alpha diversity indices (Shannon, Simpson, Chao1, and Good’s coverage) were determined by QIIME (version 1.9.1). For beta diversity index, the principal co-ordinates analysis (PCoA) analysis was performed by ade4 package and ggplot2 package in R software (Version 2.15.3; R Foundation for Statistical Computing, Vienna, Austria).

### Statistical analysis

Microbial populations were exhibited as cfu/g FM and transformed to log10 prior to statistical analysis. The collected data were subjected to the GLM procedure in SPSS Statistics (version 23.0; IBM, Armonk, NY, USA). The detailed model for fermentative characteristics of mixed silages was: Y = μ+C^i^+L_j_+D_k_+C_i_L_j_+C_i_D_k_+L_j_D_k_+C_i_L_j_D_k_+ɛ_ijkl_, where Y is observation, μ is the general mean, C_i_ is the effect of cellulase addition (i = 2, with and without), L_j_ is the effect of LAB inoculation (j = 2, with and without), D_k_ is the effect of ensiling periods (k = 7, 14, and 30 d), the C_i_L_j_, C_i_D_k_, L_j_D_k_ and C_i_L_j_D_k_ are the interactions of these factors, and the ɛ_ijkl_ is the residual error. Data on chemical composition of mixed silages was analyzed by two-way analysis of variance to evaluate the effects of cellulase, LAB and their interaction. Each plastic ensiling bag was utilized as the experimental unit. The Duncan’s test was done for multiple comparisons and the differences were declared significant when p < 0.05. The microbial community data were analyzed on the platform of Novogene (Beijing, China).

## RESULTS AND DISCUSSION

### Chemical composition before ensiling

Chemical composition before ensiling is shown in Table 1. The DM contents of WBG and CS were 19.06% FM and 88.37% FM, respectively. The DM content of raw materials is one of the most important factors affecting the microbial growth and reproduction, which greatly determines the silage quality [14]. When WBG was mixed with CS at 7:3 of FM, the DM content of the mixture was 38.31% FM, which met the minimum requirement of 30% to 35% FM for well-preserved silage [15]. The CP contents of the two materials were 27.13% and 5.46%, respectively, on a DM basis, while it was 13.38% DM after mixing. The NDF and ADF contents of WBG were lower than that of CS, while these were 58.90% and 33.26% DM in WBG+CS. The WSC content of WBG+ CS was 4.61% DM, which meets the minimum level of 3% DM of fresh materials for desirable fermentation as described by a previous study [16].

### Chemical composition of mixed silages

Chemical composition of silages after 30 d of ensiling are shown in Table 2. No two-way interaction of C×L was noted for all the parameters, and no significant difference was found among all silages on the DM content. In the current study, when LAB was added alone or together with cellulase, the CP content was higher as compared to CON (p<0.05), which indicates that CP was well protected from intensive proteolysis by undesirable microbes [17]. This could be verified by the fact that CON had the highest NH_3_-N concentration (Table 3). Application of cellulase (CEL and CLA) decreased the NDF and ADF contents (p<0.05). It could be speculated that cellulase directly hydrolyzed the structural carbohydrates (cellulose and hemicellulose) into glucose, which was possibly utilized for LAB proliferation and growth. Similar results have been previously reported [3,18]. LAB-inoculated silage did not show reductions of the NDF and ADF contents, which was consistent with a previous report [2]. LAB inoculation usually does not degrade fiber components, because cellulose-degrading enzymes are not generated during ensiling with LAB application [19]. Another possible reason could be that though WBG was susceptible to the pH decline and enzyme hydrolysis, the structural components contained in the rough and dry CS was not easily degraded when LAB was inoculated separately. No significant difference on WSC was found among all silages. This was consistent with several previous studies, including those with soybean residue and corn stover mixed silage [3] and high-moisture amaranth and rice straw mixed silage with cellulase and LAB application [20]. However, it did not match the results of a study which used corn and hulless barely straw mixed silage [18], where more WSC were found to be generated with cellulase addition and LAB inoculation on d 30 of ensiling due to the inhibited fermentation of undesired bacteria. The discrepancy could be due to the different type and chemical composition of the ensiling materials and varying experimental environment. Considering microbial populations, there was a C×L two-way interaction on LAB counts (p<0.001). All the treated groups showed higher LAB populations than CON (p< 0.001). The cellulase addition possibly raised the LAB counts, as degradation of the cell wall fractions generated more WSC, which promoted LAB proliferation and growth [21]. However, no *E. coli* and mold were detected in the present study even in the CON group, which indicated that all ensiling bags were sealed adequately without any aerobic exposure. Moreover, undesirable deterioration caused by spoilage-inducing microbes during ensiling were greatly inhibited in the present study, which could also be ascribed to the low pH (<3.89) (Table 3) after 30 d of ensiling. Yeast is one of undesirable microorganisms which may cause secondary fermentation. In this study, yeast was below the detection level in CEL and LAB silages (less than 2.00 log10 cfu/g of FM) and absent in CLA. However, the yeast count in CON silage was 4.28 log10 cfu/g of FM. Compared to CON silage, the lower level of undesirable yeast counts in treated silages could be partly explained by the higher LAB counts. Antimicrobial metabolites generated from LAB fermentation, like bacteriocins and tannins, blocked the spoilage-inducing microorganism. Moreover, when competing for common nutritional substrates with the harmful microbes, LAB dominated the ensiling process from the early stage [22].

### Fermentation dynamics and characteristics of mixed silages

Considering the pH assessment during ensiling in the current study, both pH dynamics and the final pH value showed good trends. There were two-way interactions between C×L and C×D for the pH value (p<0.001). LAB and CLA had sharper decline as compared to CON and CEL (p<0.001) until they reached their lowest value within 7 d. All additive-treated groups showed lower pH than CON on day 30 (p< 0.001), with all groups showing pH less than 4.2, which meets the requirement for good silage [23]. Therefore, both cellulase and LAB accelerated the pH decline, with the decline being accelerated when LAB was added (LAB and CLA). These results could explain the high LAB counts and low undesirable microbial counts (Table 2).

There were 3-way interactions among C×L×D for the LA concentration (p = 0.030), AA concentration (p = 0.048), and the LA/AA ratio (p<0.001). The LA concentration accumulated gradually with the increasing ensiling period prolonging until d 30. On d 30 of ensiling, the CLA silage showed higher LA production than other groups (p<0.001), while it did not increase significantly in CEL as compared to CON. This indicated that the combined application of cellulase and LAB further increased the LA production as compared to silage separately treated with cellulase and LAB.

Similar to the LA concentration, the AA concentration in all the groups also accumulated during the prolonged ensiling period. However, the highest AA concentration was found in the CON silage after 7 d of treatment. This might be due to the lack of sugar as the forage WSC was constantly consumed with the prolonged fermentation. LAB fermentation usually shifts to being heterofermentative under low-WSC conditions or in forage where sugar is insufficient, like in legumes and tropical grasses, despite LAB fermentation normally being homofermentative [23]. A higher AA production was also previously found in the no-additive-control of the high moisture amaranth and rice straw mixed silage in the early ensiling stage [20]. The lower WSC concentration in the CON, as compared to other groups, promoted the shift from homofermentation towards heterofermentation, thus generating the highest AA concentration in CON after 30 d of storage in the current study. PA and BA were not detected in all groups during ensiling. This might be due to the low pH condition in the present study. As the pH went below 4.31 from d 7 of ensiling process, it created an acidic environment that inhibited the PA-producing bacteria and the clostridial fermentation process, which generates BA via protein degradation. The LA/AA ratio was its highest value on d 14, with the CON silage being lower value than others (p<0.001).

In the current study, the addition of cellulase and LAB significantly decreased the NH_3_-N content (p<0.05) as compared to CON, with all groups showing low NH_3_-N content (<6% of total nitrogen [TN]), which meets the requirement for high-quality silage (<10% of TN) [24]. This could be due to the low pH during ensiling in all groups and proper DM content (38.31% DM) in fresh materials. As previous studies [15,25] have shown, the NH_3_-N content is related to plant protease, clostridia, or enterobacteria, with high NH_3_-N production indicating excessive proteolysis during the ensiling process, which might be due to the slow pH decline, especially during the early fermentation stage and clostridial fermentation. Clostridial activities in silage can result in losses of DM and nutrient, as they utilize WSC, proteins, or LA as substrates. Additionally, BA production might also increase during the process [15]. However, clostridia are susceptible to the high DM content in silage, with ensiling materials containing more than 30% DM reportedly inhibiting the clostridial activities [15]. In this study, the absence of clostridia and BA supported the current study result of low NH_3_-N content. Similar results about NH_3_-N reduction caused by cellulase and LAB application have been previously reported [18,20].

### Venn analysis and alpha diversity indices of microbial community

**Figure 1:**
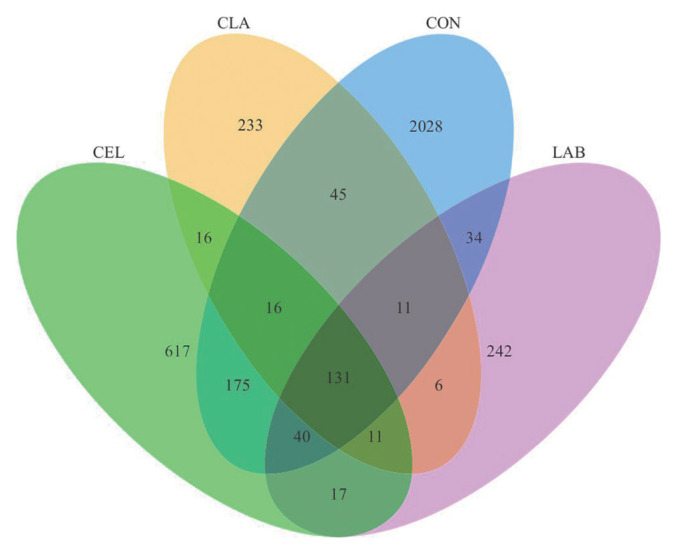
Venn analysis of OTUs in silages of d 30. OTUs, operational taxonomic units; FM, fresh matter; CON, no additives; CEL, added cellulase (120 U/g FM); LAB, added lactic acid bacteria (2×10^6^ cfu/g FM); CLA, added cellulase (120 U/g FM) and lactic acid bacteria (2×10^6^ cfu/g FM).

The Venn analysis showed the shared and unique OTUs among all groups (Figure 1). Here, 2,028, 617, 242, and 233 unique OTUs were found in CON, CEL, LAB, and CLA silages, respectively, with 131 being shared among all. Unique OTUs are known to be possibly due to the quality differences in silage among the different groups. A total of 968,275 raw sequences were generated by the high-throughput amplicon sequencing of the V3–V4 hypervariable region in *16S rRNA* gene. After low quality and short sequence filtering, 796,400 qualified sequences ranging from 62,243 to 68,403 for each treatment with an average of 66,367 were obtained (Table 4). Based on a 97% similarity level, these reads were subsequently clustered into 2,968 OTUs for further analysis. The CON silage showed the highest number of OTUs, with the inoculation of LAB (LAB and CLA) exerting a bigger effect on the number of OTUs than those with cellulase application (CEL), as the CLA silage showed the lowest OTUs number. The number of observed species ranged from 224 (CLA) to 999 (CON). These findings might be attributed to the quicker pH decline and oxygen consumption in the treated groups, which limited the growth of undesirable microorganisms that could not tolerate the acid and anaerobic environment. Contrastingly, the inhibition in CON silage was weaker due to the slower formation of low pH and anaerobic conditions. Additionally, as the fermentation proceeded, LAB in silages with additives began growing rapidly, which subsequently replaced the unfavorable epiphytic microbes in raw materials and dominated the fermentation process, especially in LAB-inoculated silages (LAB and CLA). This resulted in a lower number of OTUs and observed species as compared to the CON silage, specifically the combined application of cellulase and LAB exerting the biggest effect. These results were also supported by the Shannon, Simpson, and Chao1 indices, which were the highest in CON silage, thus indicating that additive application decreased the microbial diversity and richness in silage. Similar observations were also reported in a previous study [20]. The coverages of all silage samples were higher than 99%, which showed that the sequencing data was sufficiently large to reliably capture the entire profile of the silage microbial community. Therefore, the silages with additives applied, especially LAB (LAB and CLA) had lower microbial diversity and richness.

### Relative abundance on phylum level and genus level

**Figure 2:**
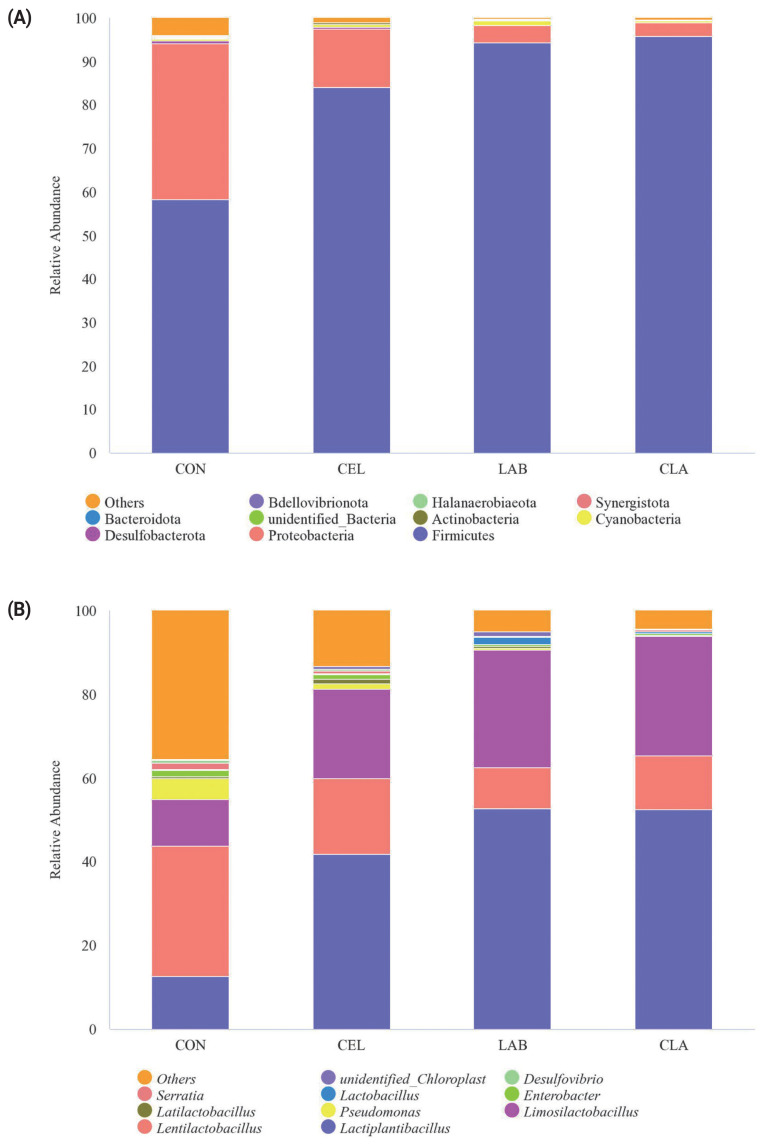
Relative abundance of microbial community on phylum level (A) and genus level (B) in silage of d 30. FM, fresh matter; CON, no additives; CEL, added cellulase (120 U/g FM); LAB, added lactic acid bacteria (2×10^6^ cfu/g FM); CLA, added cellulase (120 U/g FM) and lactic acid bacteria (2×10^6^ cfu/g FM).

On the phylum level, Firmicutes, Proteobacteria, Desulfobacterota, Cyanobacteria, and Actinobacteria were the top five dominant phyla in all silages (Figure 2). Among these, Firmicutes was the most abundant, accounting for 58.27% (CON), 84.03% (CEL), 94.31% (LAB), and 95.77% (CLA) respectively, in the four groups. In contrast, Proteobacteria accounted for 35.88% (CON), 13.51% (CEL), 4.01% (LAB), and 3.27% (CLA) in this study. Firmicutes are very important acid hydrolytic microorganisms that can proliferate and secrete numerous extracellular enzymes, including cellulases, proteases, and lipases under the low pH and anaerobic conditions during ensiling [26]. The increased abundance of Firmicutes in the additives treated groups versus CON indicated a favorable change in the fermentation quality, and this was consistent with previous studies [7,15,20]. Proteobacteria have a major role in polysaccharide utilization or BA fermentation, organic matter digestion, and carbon and nitrogen cycling in anaerobic fermentation [27]. In the current study, the CON silage showed the most abundant Proteobacteria as compared to treated groups, with the increase of the dominant phylum Firmicutes/Proteobacteria ratio from 1.62 (CON) to 6.22 (CEL), 23.54 (LAB), and 29.32 (CLA) indicating a higher silage quality in the treated groups, which were attributed to the lower pH in these silages. Although Desulfobacterota was detected in the CON and CEL silages, their abundance was less than 1%. Moreover, the detailed mechanisms and dynamics of these bacteria during silage fermentation are still not clear due to insufficient reporting till date.

Further analysis of the microbial community at the genus level is also shown (Figure 1). At the genus level, silages were highly dominated by the LAB genera containing *Lactiplantibacillus*, *Lentilactobacillus*, *Limosilactobacillus*, *Pseudomonas*, and *Latilactobacillus*. In the CON silage, *Lentilactobacillus*, *Lactiplantibacillus*, and *Limosilactobacillus* were the top three genera with abundances of 31.24%, 12.53%, and 11.06%, respectively. However, in the additives treated groups, *Lactiplantibacillus* became the predominant genus, followed by *Limosilactobacillus* and *Lentilactobacillus*. *Lactiplantibacillus* is a rod-shaped LAB which produces LA by degrading plant carbohydrates, which reduces the pH value in silage fermentation. In this study, high abundance of these genera was the direct result of the exogenous LAB inoculation of *Lactiplantibacillus plantarum* and *Lentilactobacillus buchneri*. All the treated groups, especially the LAB inoculated silages (LAB and CLA) showed higher abundance of *Lactiplantibacillus* than CON, which was consistent with a previous study [28], where the highest abundance of *Lactiplantibacillus* was found in the LAB-inoculated oat silage. These observations also explained why these treated groups had higher LA concentration and lower pH value (Table 3). According to different fermentation patterns, the LAB could be classified into homofermentative and heterofermentative types. Homofermentative LAB usually dominates fermentation by rapid LA production and sharply reducing the pH, which inhibits the growth of undesirable microorganisms. By contrast, heterofermentative LAB dominates fermentation by fermenting LA to AA and 1,2-propanediol, thus showing improved aerobic stability of silage [29]. *Lactiplantibacillus* and *Lentilactobacillus* are the two most common LAB genera for two fermentation types, respectively. In the current study, the abundance of *Lentilactobacillus* decreased by 42.48% (CEL), 68.94% (LAB), and 59.20% (CLA) respectively when compared to CON, which indicated that silages shifted from being homofermentative to heterofermentative post additives application, especially in the LAB and CLA silages. This speculation could be well corroborated by the high LA and low AA concentration in the additives treated groups (Table 3). The *Limosilactobacillus* abundance increased by 93.06%, 153.95%, and 158.48%, compared with CON here. However, limited information exists about *Limosilactobacillus* in silage and its utilization in commercial inoculants. Based on our study results, we hypothesized that similar with the well-known *Lactiplantibacillus*, *Limosilactobacillus* is also a homofermentative LAB genus, but the detailed characteristics and mechanisms of this genus needs further study. *Lactobacillus* and *Enterobacter* were extensively reported as the two most abundant genus in previous studies [20,27,30], however, in the current study they were below 2%. The distinctions might be probably due to the different ensiling materials, ensiling duration, different geographical location, etc. All these results could also be verified by the high LA production and low pH value in the treated groups, especially the CLA silage.

### Beta diversity

**Figure 3:**
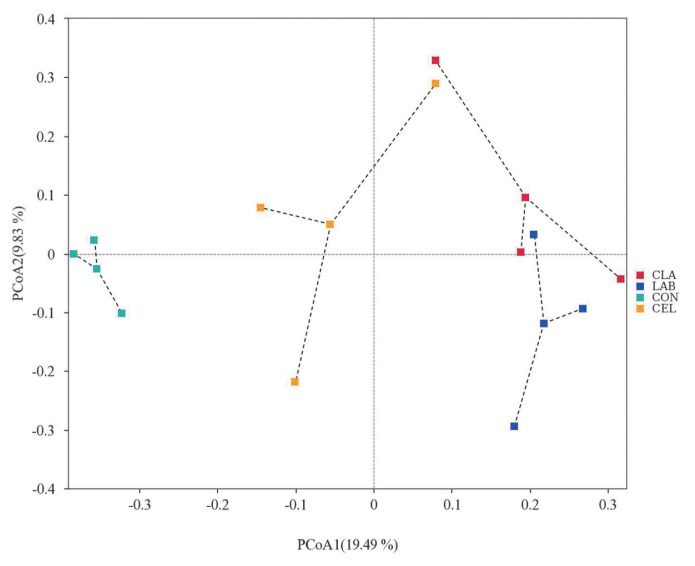
Principal co-ordinates analysis (PCoA) analysis of microbial community in silage of d 30. FM, fresh matter; CON, no additives; CEL, added cellulase (120 U/g FM); LAB, added lactic acid bacteria (2×10^6^ cfu/g FM); CLA, added cellulase (120 U/g FM) and lactic acid bacteria (2×10^6^ cfu/g FM).

Beta diversity was analyzed using PCoA to further investigate the microbial community differences among all silages (Figure 3). It was reported that silages with distinct microbial communities exhibited a tendency to separate from one another, whereas those with similar communities showed a propensity to cluster together [7]. As illustrated in the PCoA diagram, the plots of CON and CEL were clearly separated from those of LAB and CLA, whereas the distribution of PCoA plots between LAB and CLA was not separated clearly. Based on the plot distinctions among different silages, we hypothesized that the CON silage showed a different microbial community as compared with CEL, LAB, and CLA silages. These observations might help to explain the variation of silage fermentation quality in the current study.

## CONCLUSION

This study showed that mixed ensiling of WBG and CS with cellulase and LAB application is an effective preservation approach to improve the nutritional value and fermentation quality, as all the additive-treated groups showed better quality and lower microbial diversity than CON. The addition of cellulase significantly decreased the pH, NDF, ADF, and NH_3_-N contents, while increasing the LAB counts. Moreover, the inoculation of LAB significantly increased the CP, LAB counts, LA, and LA/AA, and decreased the pH, AA, and NH_3_-N content. According to the microbial community analysis, the silages inoculated with LAB showed a higher abundance of the desired Firmicutes and *Lactiplantibacillus*, and lower microbial diversity, while the cellulase addition exerted a relatively less positive effect than LAB inoculation on the microbial community parameters. Therefore, this study recommends the combined application of cellulase and LAB on the WBG-CS mixed silage, thus providing a new approach for the proper preservation and utilization of the WBG and CS agro-byproducts.

## Figures and Tables

**Table 1:** Chemical composition of forage before ensiling

Item	WBG	CS	WBG+CS^1)^
DM (% of FM)	19.06	88.37	38.31
CP (% of DM)	27.13	5.46	13.38
NDF (% of DM)	47.42	65.87	58.90
ADF (% of DM)	25.78	36.95	33.26
WSC (% of DM)	5.51	4.35	4.61
EE (% of DM)	4.33	5.83	5.43
Ash (% of DM)	7.80	6.37	6.62

WBG, wet brewer’s grains; CS, corn stover; DM, dry matter; FM, fresh matter; CP, crude protein; NDF, neutral detergent fiber; ADF, acid detergent fiber; WSC, water-soluble carbohydrate; EE, ether extracts.

1) WBG+CS, 70% WBG+30% CS.

**Table 2:** Chemical composition of mixed silages after 30 d of ensiling

Item	Treatment^1)^				SEM	p-value^2)^		
	
	CON	CEL	LAB	CLA		C	L	C×L
Chemical compositions (% of DM)								
DM (%)	37.16	37.88	37.77	37.96	0.184	0.249	0.378	0.494
CP	11.84^b^	12.37^ab^	12.75^a^	13.06^a^	0.130	0.143	0.015	0.676
NDF	56.05^a^	54.12^b^	55.25^a^	53.93^b^	0.136	<0.001	0.106	0.29
ADF	32.41^a^	31.28^bc^	31.94^ab^	30.96^c^	0.110	0.001	0.11	0.732
WSC	0.70	0.75	0.70	0.70	0.009	0.232	0.193	0.147
Microbial population (log10 cfu/g of FM)								
LAB	8.41^c^	8.74^b^	8.87^a^	8.89^a^	0.007	<0.001	<0.001	<0.001
*E. coli*	ND	ND	ND	ND	-	-		
Mold	ND	ND	ND	ND	-	-		
Yeast	4.28	<2.00	<2.00	ND	-	-		

SEM, standard error of means; DM, dry matter; CP, crude protein; NDF, neutral detergent fiber; ADF, acid detergent fiber; WSC, water-soluble carbohydrate; LAB, lactic acid bacteria; cfu, colony-forming unit, FM, fresh matter; ND, not detected.

1) CON, no additives; CEL, added cellulase (120 U/g FM); LAB, added lactic acid bacteria (2×10^6^ cfu/g FM); CLA, added cellulase (120 U/g FM) and lactic acid bacteria (2×10^6^ cfu/g FM).

2) C, cellulase; L, lactic acid bacteria; C×L, interaction between cellulase and lactic acid bacteria.

a–c Means within a row followed by different lowercase superscripts differ (p<0.05).

**Table 3:** Fermentative dynamics and characteristics of mixed silages after 30 d of ensiling

Item	Ensiling periods (d)	Treatment^1)^				SEM	p-value^2)^					
	
		CON	CEL	LAB	CLA		C	L	C×L	D		
pH	7	4.31^Aa^	4.19^Ab^	3.74^c^	3.73^c^	0.005	<0.001	<0.001	<0.001	<0.001	C×D	<0.001
	14	4.02^Ba^	3.89^Bb^	3.73^c^	3.73^c^						L×D	0.710
	30	3.89^Ca^	3.80^Bb^	3.75^c^	3.74^c^						C×L×D	0.637
LA (% of DM)	7	0.77^Cb^	0.83^Bb^	1.84^Ba^	1.95^Ba^	0.013	<0.001	<0.001	0.913	<0.001	C×D	<0.001
	14	1.59^Bc^	1.86^Ab^	2.53^Aa^	2.59^Aa^						L×D	0.491
	30	1.82^Ac^	1.88^Ac^	2.56^Ab^	2.77^Aa^						C×L×D	0.030
AA (% of DM)	7	0.53^C^	0.53^C^	0.53^B^	0.51^B^	0.004	<0.001	0.011	0.507	<0.001	C×D	0.005
	14	0.68^Ba^	0.66^Bab^	0.63^Abc^	0.61^Ac^						L×D	0.428
	30	0.77^Aa^	0.71^Aab^	0.66^Ab^	0.66^Ab^						C×L×D	0.048
LA/AA	7	1.47^Bb^	1.56^Bb^	3.50^a^	3.87^a^	0.035	<0.001	<0.001	0.877	<0.001	C×D	0.001
	14	2.35^Ac^	2.84^Ab^	4.00^a^	4.26^a^						L×D	0.714
	30	2.35^Ab^	2.66^Ab^	3.89^a^	4.21^a^						C×L×D	0.371
NH_3_-N (% of TN)	7	2.47^C^	2.39^C^	2.41^B^	2.43^B^	0.027	<0.001	0.007	0.004	<0.001	C×D	<0.001
	14	4.11^Ba^	3.82^Bb^	3.78^Ab^	3.79^Ab^						L×D	0.143
	30	5.61^Aa^	5.00^Ab^	4.08^Ac^	4.10^Ac^						C×L×D	0.153

SEM, standard error of the mean; DM, dry matter; LA, Lactic acid; AA, Acetic acid; LA/AA, lactic acid: acetic acid; NH_3_-N, ammonia nitrogen; TN, total nitrogen; FM, fresh matter.

1) CON, no additives, CEL, added cellulase (120 U/g FM); LAB, added lactic acid bacteria (2×10^6^ cfu/g FM); CLA, added cellulase (120 U/g FM) and lactic acid bacteria (2×10^6^ cfu/g FM).

2) C, cellulase; L, lactic acid bacteria; D, ensiling periods; C×L, interaction between cellulase and lactic acid bacteria; C×D, interaction between cellulase and ensilage periods; L×D, interaction between lactic acid bacteria and ensilage periods; and C×L×D, 3-way interaction among cellulase, lactic acid bacteria and ensilage periods.

A–C Means within a column followed by different uppercase superscripts differ (p<0.05).

a–c Means within a row followed by different lowercase superscripts differ (p<0.05).

**Table 4:** Alpha diversity indices of microbial community in mixed silage of d 30

Item	Treatment^1)^			

	CON	CEL	LAB	CLA
Number of sequences	62,243	67,924	68,403	66,897
Number of OTUs	2,159	748	373	364
Observed species	999	422	238	224
Shannon index	3.84	2.56	2.11	1.88
Simpson index	0.84	0.69	0.63	0.60
Chao1 index	1,145.83	596.97	310.19	292.00
Goods coverage	0.993	0.996	0.998	0.998

OTUs, operational taxonomic units; FM, fresh matter.

1) CON, no additives; CEL, added cellulase (120 U/g FM); LAB, added lactic acid bacteria (2×10^6^ cfu/g FM); CLA, added cellulase (120 U/g FM) and lactic acid bacteria (2×10^6^ cfu/g FM).
